# Describing a landscape we are yet discovering

**DOI:** 10.1007/s10182-022-00449-5

**Published:** 2022-06-09

**Authors:** Sebastian Contreras, Jonas Dehning, Viola Priesemann

**Affiliations:** 1 Max Planck Institute for Dynamics and Self-Organization, Göttingen, Germany; 2 Institute for the Dynamics of Complex Systems, University of Göttingen, Göttingen, Germany

## Comment on *On the role of data, statistics and decisions in a pandemic*

At the beginning of the COVID-19 pandemic, very little was known about both the disease and the virus that caused it. As more information became available, the public awareness reached unprecedented scales; epidemiological terms such as *incidence* or *reproduction number* infected our daily conversations. Consequently, high pressure fell on the shoulders of policymakers, who were expected to point us to the way out of this global health threat. However, whom do we ask when we all are still learning? We would say, “let us build a model!”. In their manuscript, Jahn and coauthors present a comprehensive overview of models and their role in evidence-based decision-making. They categorize different models according to their purpose and illustrate how they have served different purposes in the context of the ongoing pandemic.

The COVID-19 pandemic has been characterized by fast dynamics and evolving in timescales shorter than we would like. In that way, the system we study is not precisely static. Facts that initially were uncertain, such as the value of $R_0$ and the generation interval exhibited by COVID-19, or the rapidly mutating nature of SARS-CoV-2, are now relatively well-constrained inputs to our models. Furthermore, mechanisms such as vaccines and medication have become available. Likewise, a large set of non-pharmaceutical interventions can be implemented or recommended by governmental entities. All this changes the course of the pandemic. So, how should we proceed to model the new pandemic if we know that our modeling framework and parameters will undoubtedly change?

As summarized by the authors, models (as a broad concept) can be used to predict, explain, and support decisions. Models can cast current (nowcast) or future (forecast) trends on parameters of interest, can help determine which parameters strongly impact particular phenomena, and how different interventions could help vary these parameters and thereby shift the observed regime. The question is then, how models can unleash their full potential. In our perspective, two factors determine the usability of a model: (i) data quality and (ii) the mechanisms we explicitly incorporate into the model. However, neither data (data-driven models) nor theoretical models alone are enough to assess the risks and impacts of a pandemic. On the one hand, purely data-driven approaches (as ML models) fail to describe what they have not seen in data, and, on the other hand, purely theoretical models fail to exhibit those effects that have not been explicitly incorporated into their structure—and perhaps this is also the reason why AI-driven models have performed so underwhelmingly. To be helpful in a fast-changing environment, models have to evolve with the phenomena they aim to describe.

The more we know about the relevant parameters, and the better we incorporate them into the model, the more the models can tell us. Here, we want to put a particular focus on Bayesian models: As we continuously update our knowledge about the pandemic and its evolution, Bayesian models naturally enable us to integrate that knowledge into our predictions and thus have found broad application in the context of the COVID-19 pandemic. They have proven useful to infer the effectiveness of interventions (Brauner et al. 2020; Sharma et al. 2021; Dehning et al. 2020), the spreading rates of SARS-CoV-2 variants (Oróstica et al. 2021), and how lethal they are (Mena et al. 2021). However, for our models to age well, we must systematically reassess them and confirm that we explicitly include all relevant mechanisms. We need surveillance to check when our assumptions start diverting from reality, continuously monitoring whether our models still capture all relevant features and timescales. This requires interdisciplinary collaboration; we need to study whether behavioral changes among the population have modified contact patterns (and thus the way diseases spread) and how these changes relate to (mis)information and fear (Dönges et al. 2022; Epstein et al. 2021; Teslya et al. 2020; Wang et al. 2019; Funk et al. 2009). In practice, we need to know which are the dominant variants spreading among the population in a given time (Oróstica et al. 2022, 2021) so that we get our estimates for the reproduction number right (and the requirements for isolation and contact reduction!). We must pay attention to official testing criteria and the asymptomatic fraction of cases, so we calculate testing requirements right (Contreras et al. 2021). We need psychological studies applied to communication to raise vaccine willingness (in other words, to reduce vaccine hesitancy) to the levels required to achieve the theoretical population immunity threshold (Lobinska et al. 2022; Shingai and Charles Shey 2021), and models to assess vaccination dynamics (Wagner et al. 2022; Crellen et al. 2021). Finally, we need to monitor signals of pandemic fatigue that could compromise the effectiveness of interventions (Petherick et al. 2021).

To sum up, we need an interdisciplinary approach to combine data and modeling in a way so versatile that we can promptly adapt to the changing conditions and environments. To prepare for the next pandemic, we need to start talking early on and foresee the relevant phenomena that will accompany it. As for COVID-19, mitigation and vaccination progress were hampered by infodemics. A future pandemic could be accompanied by migrations induced by climate changes and pathogens thriving in these new conditions. Thus, together with experts across disciplines (Iftekhar et al. 2021; Oliu-Barton et al. 2022), we will need to combine both models and data, as it forces us to structure and challenge our knowledge about the crisis. A model distills contradictions and forces one to make quantitative statements instead of (often polarizing) qualitative ones. We will need again to distill what consensus is across disciplines and where uncertainties or differences prevail (Priesemann et al. 2020, 2021). Paying attention to a single approach or a single discipline is to forego part of our knowledge voluntarily. Models are thus the backbone that structures trans-disciplinary exchange.
